# The TMEM106B risk allele is associated with lower cortical volumes in a clinically diagnosed frontotemporal dementia cohort

**DOI:** 10.1136/jnnp-2017-315641

**Published:** 2017-04-26

**Authors:** Sophie R Harding, Martina Bocchetta, Elizabeth Gordon, David M Cash, M Jorge Cardoso, Ron Druyeh, Sebastian Ourselin, Jason D Warren, Simon Mead, Jonathan D Rohrer

**Affiliations:** 1 Dementia Research Centre, Department of Neurodegenerative Disease, UCL Institute of Neurology, Queen Square, London, UK; 2 Translational Imaging Group, Centre for Medical Image Computing (CMIC), University College London, London, UK; 3 MRC Prion Unit, Department of Neurodegenerative Disease, UCL Institute of Neurology, Queen Square, London, UK

**Keywords:** FRONTOTEMPORAL DEMENTIA, PRIMARY PROGRESSIVE APHASIA

## Introduction

Frontotemporal dementia (FTD) is a neurodegenerative disease associated with impaired behaviour, language and motor function. Around a third of FTD is familial, with mutations in microtubule-associated protein tau (*MAPT*), progranulin (*GRN*) and chromosome 9 open reading frame 72 (*C9orf72*) being the most common genetic causes. No other risk factors for FTD had been identified until a genome-wide association study of patients with the most common pathological form of FTD, frontotemporal lobar degeneration with TDP-43 inclusions (FTLD-TDP), showed an association with single nucleotide polymorphisms (SNPs) in a region encoding transmembrane protein 106b (TMEM106b).^1^ For the rs1990622 SNP the minor G allele was found to be less common in patients with FTLD-TDP than in a healthy control population, that is, those expressing this allele were less likely to develop disease, and this effect was greatest in those with *GRN* mutations (who have FTLD-TDP pathology).^1^ Further studies of FTD cohorts have replicated the finding in other *GRN* cohorts, in those with *C9orf72* expansions (who also have FTLD-TDP pathology) and in one undifferentiated clinical FTD cohort.^2 3^ Furthermore, neuroimaging studies have shown reduced left hemispheric grey matter volume in those carrying the major (risk) A allele within a healthy population cohort,^4^ and reduced functional connectivity in those homozygous for the risk allele in a *GRN* cohort.^5^ In this study we investigate the association of the rs1990622 SNP with cortical volumes in a clinically diagnosed FTD cohort and a subset of cases with likely FTLD-TDP.

## Methods

The UCL FTD DNA cohort database was searched for those with volumetric MRI. Data were available from 198 patients with an FTD spectrum disorder: 87 with behavioural variant FTD (bvFTD), 46 with non-fluent variant primary progressive aphasia (nfvPPA), 43 with semantic variant PPA (svPPA), 6 with a primary progressive aphasia (PPA) syndrome not meeting criteria for any of the other PPA syndromes (called PPA-not otherwise specified here, abbreviated to PPA-NOS), 5 with FTD associated with motor neurone disease (FTD-MND), 7 with corticobasal syndrome (CBS) and 4 with progressive supranuclear palsy (PSP). Within this cohort, 76 individuals had definite or likely TDP-43 pathology: this group included cases with genetic mutations known to cause TDP-43 pathology (*GRN* (n=10), *C9orf72* (n=17), dual *GRN/C9orf72* (n=1), *TBK1* (n=1) and *SQSTM1* (n=2) mutations) and those with clinical syndromes associated with FTLD-TDP (FTD-MND and svPPA). Genotyping of the rs1990622 polymorphism in the total FTD cohort showed a frequency of AA 77 (38.9%: 31 bvFTD, 19 nfvPPA, 20 svPPA, 1 PPA-NOS, 1 FTD-MND, 2 CBS, 3 PSP), AG 90 (45.5%: 42 bvFTD, 21 nfvPPA, 17 svPPA, 2 PPA-NOS, 3 FTD-MND, 4 CBS, 1 PSP) and GG 31 (15.7%: 14 bvFTD, 6 nfvPPA, 6 svPPA, 3 PPA-NOS, 1 FTD-MND, 1 CBS, 0 PSP), and in the TDP-43 subset of AA 33 (43.4%), AG 34 (44.7%) and GG 9 (11.8%). The frequency within a UK control population of 5020 people from the Wellcome Trust Case Control Consortium was AA 1637 (32.6%), AG 2485 (49.5%) and GG 898 (17.9%). Using a homozygous protective allele model, there was no difference in the TDP-43 subset (Fisher’s exact test, p=0.225) or the total FTD cohort (Fisher’s exact test, p=0.450) compared with the control group, nor did allelic tests show significant differences between patients and controls. However, a homozygous risk allele model in the TDP-43 subset gave Fisher’s exact test, p=0.049 compared with the control group, and in the total FTD cohort gave Fisher’s exact test, p=0.076 compared with the control group.

Cortical grey matter volumes (corrected for total intracranial volume) for the insula, cingulate, frontal, temporal, parietal and occipital lobes in each hemisphere were generated from volumetric T1 MRI using a previously described methodology.^6^ Given the previous imaging studies and the results of the models above,^4 5^ volumes were compared between the homozygous risk allele group (AA) and those carrying at least one protective allele (AG/GG group) using non-parametric tests (Mann-Whitney U). These two groups did not significantly differ in age at MRI scan (total FTD cohort—AA: mean 63.6 (SD 7.3) years, AG/GG: 63.2 (9.2); TDP-43 subset—AA: mean 62.2 (6.7) years, AG/GG: 63.9 (7.2)) or disease duration at time of MRI scan (total FTD cohort—AA: mean 4.9 (2.4) years, AG/GG: 4.9 (3.4); TDP-43 subset—AA: mean 4.9 (2.2) years, AG/GG: 5.0 (3.0)).

## Results

In the total FTD cohort, significantly lower volumes were present in the AA (risk, major allele) group compared with the AG/GG group in the frontal (p=0.009), temporal (p=0.029), cingulate (p=0.014) and insula (p=0.018) cortices (table 1).

**Table 1:** Mean (standard deviation) cortical volumes (whole brain and each hemisphere) as a percentage of total intracranial volume in the total FTD cohort and TDP-43 subset

	**Frontal**	**Temporal**	**Parietal**	**Occipital**	**Cingulate**	**Insula**
**Total FTD cohort: whole brain**						
AA	9.3 (0.9)	6.1 (0.8)	5.5 (0.4)	4.4 (0.4)	1.44 (0.14)	0.69 (0.12)
AG/GG	9.6 (1.0)	6.3 (0.8)	5.4 (0.6)	4.4 (0.4)	1.49 (0.14)	0.73 (0.11)
**Total FTD cohort: left hemisphere**						
AA	4.6 (0.5)	2.9 (0.5)	2.7 (0.3)	2.2 (0.2)	0.68 (0.09)	0.34 (0.06)
AG/GG	4.8 (0.5)	3.0 (0.5)	2.7 (0.3)	2.2 (0.2)	0.72 (0.08)	0.36 (0.06)
**Total FTD cohort: right hemisphere**						
AA	4.7 (0.5)	3.2 (0.4)	2.8 (0.2)	2.2 (0.2)	0.76 (0.08)	0.36 (0.07)
AG/GG	4.8 (0.6)	3.2 (0.4)	2.7 (0.3)	2.2 (0.2)	0.77 (0.09)	0.37 (0.06)
**TDP-43 subset: whole brain**						
AA	9.4 (1.0)	5.8 (0.9)	5.4 (0.5)	4.4 (0.4)	1.42 (0.16)	0.69 (0.08)
AG/GG	9.8 (1.0)	6.0 (0.9)	5.5 (0.4)	4.4 (0.3)	1.49 (0.14)	0.72 (0.10)
**TDP-43 subset: left hemisphere**						
AA	4.6 (0.5)	2.7 (0.6)	2.6 (0.3)	2.2 (0.2)	0.66 (0.10)	0.33 (0.05)
AG/GG	4.9 (0.5)	2.8 (0.6)	2.8 (0.3)	2.2 (0.2)	0.69 (0.08)	0.35 (0.06)
**TDP-43 subset: right hemisphere**						
AA	4.8 (0.5)	3.1 (0.4)	2.8 (0.3)	2.2 (0.2)	0.76 (0.08)	0.36 (0.05)
AG/GG	4.9 (0.5)	3.2 (0.4)	2.8 (0.3)	2.2 (0.1)	0.79 (0.09)	0.37 (0.05)

FTD, frontotemporal dementia.

As previous studies have shown an association of the rs1990622 polymorphism with decreased volumes in the left hemisphere,^4^ further analysis of cortical volumes within each hemisphere was performed. Significant differences between the two groups were shown only in the left hemisphere: in the frontal (p=0.003), temporal (p=0.019), cingulate (p=0.004) and insula (p=0.004) cortices (table 1).

In the TDP-43 subset, although similar or larger absolute differences in volume were seen in the AA group compared with the AG/GG group, a significant difference was only seen between the groups in the frontal lobe (p=0.044), with a trend to a difference in the cingulate (p=0.085) and insula (p=0.084) cortices. As with the clinical cohort, this was driven by an effect within the left hemisphere, with significant differences seen between groups in the frontal (p=0.013) and parietal (p=0.037) cortices, and a trend to a difference in the cingulate (p=0.082) and insula (p=0.072) (table 1).

## Discussion

In a cohort of patients clinically diagnosed with FTD and also in a subset of patients with likely TDP-43 pathology, we show that homozygosity for the TMEM106B risk allele is associated with reduced grey matter volume in key cortical regions implicated in FTD. Consistent with previous research showing an effect of the TMEM106B risk allele on left hemisphere structures within the general population, decreased grey matter volumes were found in the left but not right hemisphere in both the total cohort and the TDP-43 subset.

Despite some studies showing no effect, overall, previous studies have shown that the effects of TMEM106B variants are seen in cases of FTD with FTLD-TDP (but not FTLD-tau), including those with mutations in *GRN* or *C9orf72,* as well as those without.^2^ Investigation of clinically diagnosed cohorts has been variable, with only one study showing a positive effect. This variability is most likely to represent stronger effects in the gene mutation carriers and because of variable extent of TDP-43 pathology within each clinical cohort, with a greater number of FTLD-TDP cases resulting in a significant association within a cohort. In the cohort described here, at least 76/198 cases have probable or definite TDP-43 pathology, but this number is likely to be much higher as a substantial proportion of bvFTD cases and a smaller proportion of nfvPPA/PPA-NOS cases will also have FTLD-TDP, hence the positive imaging findings in this study. Ideally, studies should be performed in postmortem confirmed cohorts, but in such a rare disease this is difficult.

TMEM106B is a glycoprotein critical for normal lysosomal function.^2^ Although its exact role in the pathogenesis of FTD remains unclear, studies have shown that TMEM106B appears to have an effect on the pathological burden of TDP-43 pathology.^2^ It is unsurprising then that cell loss measurable as cortical grey matter atrophy on MRI is associated with the TMEM106B polymorphism. However further work is needed to understand the exact relationship and the extent of the effect in different pathological and genetic subtypes of FTD.
